# Fungal Community Structure in Disease Suppressive Soils Assessed by 28S LSU Gene Sequencing

**DOI:** 10.1371/journal.pone.0093893

**Published:** 2014-04-03

**Authors:** C. Ryan Penton, V. V. S. R. Gupta, James M. Tiedje, Stephen M. Neate, Kathy Ophel-Keller, Michael Gillings, Paul Harvey, Amanda Pham, David K. Roget

**Affiliations:** 1 Center for Microbial Ecology, Michigan State University, East Lansing, Michigan, United States of America; 2 CSIRO Ecosystem Sciences, Glen Osmond, South Australia, Australia; 3 Department of Agriculture, Fisheries and Forestry, Queensland, Leslie Research Centre, Towoomba, Queensland, Australia; 4 SARDI, Glen Osmond, South Australia, Australia; 5 Department of Biological Sciences, Macquarie University, North Ryde, New South Wales, Australia; Virginia Tech, United States of America

## Abstract

Natural biological suppression of soil-borne diseases is a function of the activity and composition of soil microbial communities. Soil microbe and phytopathogen interactions can occur prior to crop sowing and/or in the rhizosphere, subsequently influencing both plant growth and productivity. Research on suppressive microbial communities has concentrated on bacteria although fungi can also influence soil-borne disease. Fungi were analyzed in co-located soils ‘suppressive’ or ‘non-suppressive’ for disease caused by *Rhizoctonia solani* AG 8 at two sites in South Australia using 454 pyrosequencing targeting the fungal 28S LSU rRNA gene. DNA was extracted from a minimum of 125 g of soil per replicate to reduce the micro-scale community variability, and from soil samples taken at sowing and from the rhizosphere at 7 weeks to cover the peak *Rhizoctonia* infection period. A total of ∼994,000 reads were classified into 917 genera covering 54% of the RDP Fungal Classifier database, a high diversity for an alkaline, low organic matter soil. Statistical analyses and community ordinations revealed significant differences in fungal community composition between suppressive and non-suppressive soil and between soil type/location. The majority of differences associated with suppressive soils were attributed to less than 40 genera including a number of endophytic species with plant pathogen suppression potentials and mycoparasites such as *Xylaria* spp. Non-suppressive soils were dominated by *Alternaria*, *Gibberella* and *Penicillum*. Pyrosequencing generated a detailed description of fungal community structure and identified candidate taxa that may influence pathogen-plant interactions in stable disease suppression.

## Introduction

Plant-microbe-soil interactions play a vital role in maintaining plant health and productivity in agricultural and horticultural crops. Plant diseases caused by soilborne pathogens result in substantial losses to agricultural production worldwide [1]–[3]. For example, roots of cereals, pasture plants and oil seed crops are prone to attack by soilborne necrotrophic pathogens such as *Rhizoctonia solani, Fusarium pseudograminearum, Gaeumannomyces graminis* var *tritici* and *Pythium* spp. These are among the most difficult groups of plant pathogens to control due to their ability to persist in crop residues [1]–[4]. Due to the limitations in the effectiveness of fungicides and a lack of successful plant-based resistance, enhancement of soil-based natural disease suppression could be an effective option to control disease, especially if it can be achieved by in-field enhancement through crop and/or soil management practices [5]–[9].

Soil suppressiveness is the ability of a soil to prevent/suppress disease even in the presence of a pathogen, suitable host plant and favorable climatic conditions [9]–[13]. In this study we use the term ‘non-suppressive’ for soils that are unable to suppress disease incidence by the pathogen. Biological suppression of soilborne pathogens has been reported from a variety of cropping systems worldwide [11]–[12], [14]–[15]. In the case of wheat and barley crops, this suppression has been shown against a number of soilborne diseases including *Fusarium* wilt, Take-all and *Rhizoctonia* bare patch. In Australia, biologically-based disease suppression has been reported in long-term experimental plots and farmer fields [11], [16]–[17]. This suppression has been attributed to diverse microbial communities including bacteria, fungi and protozoa and is reported to affect pathogen survival, growth in bulk soil and rhizosphere and root infection [18]–[19]. The adoption of no-till and stubble retention practices can, in some cases, increase soilborne plant diseases in the short-term [11]–[20]. However, long-term adoption of crop management practices that supply higher levels of biologically-available carbon inputs either through crop residues or addition of composts and organic manures can support higher levels of suppression. This occurs through changes to the composition and activity of the soil microbial community [7], [21]–[23].

Rhizoctonia bare patch disease generally starts in young seedlings and the disease manifests during the first 8 weeks of crop growth causing significant crop yield losses [4]. Two complementary mechanisms are suggested to be involved in disease suppression in both the bulk soil and rhizosphere; competition for nutrients between the pathogen and general microbial community and the activity of antagonists [7]. Interactions in bulk soil involve general competition for carbon and nutrients (fungistatis) or antibiosis (soil bacteria or fungi vs. pathogenic fungi) and mycoparasitism (pathogenic fungi vs. other soil fungi) that can affect the survival and growth of the pathogen [24]–[26]. Rhizosphere interactions can directly prevent the pathogen reaching the root or interfere with infection processes [27]. Indirectly such interactions may induce host plant resistance [14], [28].

Research on microbial communities in disease suppressive soils has mainly focused on bacteria [28]–[31]. A wide range of bacterial groups have been suggested as contributing to disease suppression through antibiosis, plant growth promotion or systemic induced resistance [13], [14], [30]. The functional diversity of soil fungi and their capacity to colonize diverse microhabitats can influence pathogen levels and play a significant role in improving plant health, e.g. *Trichoderma* spp and mycorrhizal fungi [25], [32]. The genus *Trichoderma* has been studied extensively for its biocontrol potential and a number of fungi and oomycetes are registered as biocontrol agents [33]–[34]. Soils with higher disease suppressive potential have been found to exhibit higher fungal diversity [22]. In view of the large diversity of uncultured fungi in soil, culture-independent methods are required to describe their composition and to identify community differences between soils. Recently, based on high-throughput sequencing, soils from pea fields with different degrees of disease were discriminated on the basis of their fungal communities [35].

Our objective was to determine in what way fungal communities differed between paired soils, one with long-term high disease suppression and the other with no disease suppression, at two wheat-growing locations, Avon and Minnipa, in South Australia. For comparing the fungal populations among the four sites and two sampling times, we used pyrosequencing of the 28S LSU rRNA gene in soil DNA from the four fields and RDP’s Naïve Bayesian Classifier, which provides both genus identification and taxonomic placement for otherwise unclassified sequences.

## Materials and Methods

### Site and soil descriptions

The agricultural fields studied are located in the wheat-cropping region in South Australia and have been under continuous cropping for more than 10 years. The Avon and Minnipa locations are, respectively, ‘suppressive’ (SP) (S34 13.981, E138 18.586 and S32 59.066, E135 9.424) and ‘non-suppressive’ (NSP) (S34 13 29.06, E138 19 3.66 and S32 49.955, E135 9.595) for diseases caused by soil-borne necrotrophic pathogens (e.g. *Rhizoctonia solani* AG 8, *Fusarium pseudograminearum*) in cereal crops (**Figure S1**) based on field disease measurements and glasshouse pot assays over the previous 20 years [11], [36]–[37]. The fields at Minnipa are located at the Minnipa Agricultural Centre, a collaborative research organisation in this study and the land at Avon field sites has housed CSIRO research trials for over 25 years. The Avon and Minnipa sites are 350 km apart and the SP and NSP fields are 1.1 and 0.4 km apart respectively. The soil at both sites is Luvic Calcisol and sandy or sandy loam in texture (Lithocalcic Calcarosol, [38]–[39]). Organic carbon in the SP fields ranged from 1.1–1.6% and in the NSP fields from 0.7–1.0%, clay ranged from 8–17% (**Table S1**). The biological nature of higher disease suppression in these fields has been previously established [16]–[17], [36]–[37]. The climate is Mediterranean-type, characterized by hot dry summers and cool wet winters, with an average annual rainfall of 260–300 mm. During the 3 years prior to sampling all the fields were under cereal crops though the SP fields at both sites were under continuous cropping with stubble retention and no-till practices for longer periods (>10 y) compared to the NSP fields that were under a low-input pasture-crop rotation. Other general agronomic practices such as weed control, fertilizer addition and time of sowing were similar in both the SP and NSP fields. Soils from the SP fields also showed higher microbial biomass (by 20–35%) and more particulate organic carbon (10–20% higher).

Eight samples (10 cm deep cores at sowing and rhizosphere samples at 7 weeks) were collected in 2010 and mixed to generate a composite sample for each of the eight true replicates per field, for a total of 64 samples. From each of these eight replicates, 125 g of soil was used for DNA extraction. Soils at sowing were collected in the previous year’s crop row, and at 7 weeks samples were collected from the wheat rhizosphere within the 0–10 cm depth. The rhizosphere soil was defined as the soil remaining on the root after gentle shaking; both the root and rhizosphere were used for DNA extraction. Immediately after collection, samples were stored on ice and transported to the laboratory in Adelaide. Subsamples were separated for microbial and chemical analyses and bioassay experiments.

### Disease suppression potential

Surface soils collected prior to sowing in 2010 were used to measure disease suppression potential, i.e. maximum level of disease suppression that can be observed with added pathogen inoculum and under controlled environmental conditions, using a carbon amendment assay and a soil transfer assay with wheat as a host plant [17]. During the pre-incubation in both these growth chamber assays, added pathogen inoculum was allowed to interact with the native soil microbial communities, under optimal soil moisture and temperature conditions, prior to introducing the wheat [17]. Briefly, in the carbon amendment assay, Rhizoctonia root damage was measured in response to *R. solani* AG 8 inoculum (2 x, 8 mm dia disks of colonized 1/4 strength potato dextrose agar per pot) with or without the addition of sucrose (2 g granular sucrose/300 g soil per pot), a simple carbon substrate that has been shown to accentuate suppression characteristics when added to a pre-incubation [17]. Soils were pre-incubated with or without inoculum and carbon substrate for 2 weeks prior to sowing wheat (*Triticum aestivum* cv. Yitpi) and all the pots were incubated at 10°C on a 10/14 h day-night cycle. Soil moisture in the pots was maintained at 80% field capacity by adding water at 2–3 day intervals. All experiments were harvested 4–5 weeks after seedling emergence. Roots were washed carefully and scored for disease rating (0–5 scale) and plant growth [17].

For the soil transfer assay, subsamples of field soils collected at sowing were sterilized by exposure to gamma-radiation (3 cycles of 25 rads; Steritech, Victoria) and then incubated 2 weeks at 10°C with or without *Rhizoctonia solani* AG8 as inoculum and with and without addition of 10% of the original non-sterile soil. A plant assay was then performed using a method similar to that in the carbon amendment assay and roots were scored for disease severity. Carbon substrate utilization profiles of soil microbial communities were determined using specific carbon substrates selected for Australian soils through a modified [22] Microresp method [40].

### DNA extraction

Field moist soils were stored at −20°C until lyophilized for DNA extraction. DNA was extracted from ∼125 g of soil for each sample by the Root Disease Testing Service at SARDI (Adelaide) [41]. Subsamples (aliquots) of extracted DNA were shipped to Michigan State University, USA, for fungal community sequencing. qPCR assays using rDNA (TaqMan) probe sequences specific to *Rhizoctonia solani* AG 8 and *Trichoderma* spp. were conducted [41], and quantified using a DNA standard of *R. solani* AG8 DNA (pg g^−1^ soil sample). Amounts of total fungal and bacterial DNA were quantified using group specific primers (FR 1/FF390 [42]; F968/R1378 [43] based on the QuantiTect SYBR Green PCR kit (Qiagen) and the PCR was carried out on a Strategene Maxpro3000P qPCR system.

### Fungal community profiling using T-RFLP analysis

Fungal community DNA was amplified from 14 ng of template DNA using the ITS1F.FAM forward (5′-CTTGGTCATTTAGAGGAAGTAA-3′) and ITS4R.HEX reverse (5′-TCCTCCGCTTATTGATATGC-3′) primers [44]–[45]. PCR was carried out in a 35 μL total volume using 0.4 μM of primers, 0.2 mM of dNTPs, 1x PCR buffer (Qiagen, Australia), and 4 units of HotStarTaq DNA Polymerase (Qiagen, Australia). The PCR conditions were 94°C for 1 min; 56°C for 1 min; 72°C for 1 min and 1.5 min for 35 cycles. The products were checked for size and specificity by agarose gel electrophoresis followed by purification using the MiniElute 96 UF PCR Purification Kit (Qiagen, Australia) and 100–150 ng of purified PCR product was digested for 3 h at 37°C followed by 65°C denaturation with the restriction enzymes *Alu*I and *Cfo*I. The digested DNA was purified using SigmaSpin Post-Reaction Purification Columns (Sigma, Australia) and 10 μL of the purified T-RFs were analyzed for size by the Australian Genome Research Facility (Adelaide, Australia) using capillary separation on an ABI 3730 DNA analyser with a LIZ500 size standard. TRF size and intensity data were collected using the GeneMarker analysis software (version 1.85; SoftGenetics Inc.), with a minimum cut off of 100 intensity units. Relative abundances of TRFs were calculated and normalized against the total peak height of all TRFs in the profile. TRFLP fragment data were then analyzed using the Primer6 software package (Primer-E Ltd, Plymouth, U.K.) by cluster analysis and non-metric multidimensional scaling (NMDS).

### Fungal 28S Amplification, Sequencing and Processing by RDP Classifier

Anchored by conserved regions, the 28S gene contains two hypervariable regions, denoted D1 and D2 [46]–[47]. The LR3/LR0R primer combination (http://www.biology.duke.edu/fungi/mycolab/primers.htm) was used for this study. Spanning the D1/D2 regions, these primers have been identified as the most suitable in terms of amplicon length (625 bp), resolution, and accuracy by pyrosequencing [48]. Amplification was performed per methods previously described [49] and available in supplementary information (**Text S1**). Amplicons were sequenced following Lib-L adapter ligation by Utah State University CIB Genomics Core Lab and processed using the shotgun protocol. Raw sequences were quality processed (**Text S1**) and sorted by tag through the RDP pyrosequencing pipeline (http://pyro.cme.msu.edu) and subjected to classification using the RDP naïve Bayesian fungal LSU Classifier version 1 [50] that is based on a manually archived LSU gene training set [48] at 0% “best-match” bootstrap confidence (“classification confidence”-CC) [49]. Each sample was randomly re-sampled to 4,484 sequences per sample and classifications at the phylum, class, order, family, and genus levels were treated as bins for downstream statistical analysis. Supplementary text describes sequence classification at 50% bootstrap confidence to investigate the composition of the ‘unclassified’ fungi (**Text S2**) and comparisons between the whole and resampled datasets were also performed (**Text S3**). Sequences were deposited in the European Nucleotide Archive under study accession PRJEB4037, sample accessions ERS253863-ERS253910.

### Data Analysis

We used several multi-variate statistical analyses for community comparisons, for both the TR-F data and 28S sequence data, using the PRIMER-6 software package [51], (Primer-E Ltd, 239 Plymouth, U.K.). Firstly, Hellinger transformed data (square root of relative abundance) was used to generate Bray-Curtis (+1) dissimilarity matrices and an analysis of similarity (ANOSIM) was carried out to test whether different sites harbored distinct fungal communities [52]. Non-metric multidimensional scaling (nMDS) analysis was performed using all pairwise distances between different fungal communities (Bray-Curtis distances) and the statistical significance was tested through permutational analysis of variance (PERMANOVA) [53]. The average contribution of individual sampling units (e.g. genera OTU) to the overall Bray-Curtis distances was estimated using the similarity percentage (SIMPER) analysis from the pair wise comparisons [54]. The Shannon index (H’) estimate of community richness [55] and Pielou’s evenness (J’) [56] were used for community diversity estimates. Differences in the disease incidence (root infection) between fields were compared by ANOVA analysis using Genstat (v14.2, VSN International Ltd.).

## Results

### Pathogen, microbial and disease suppression properties

Soils collected at the time of sowing from the suppressive (SP) and non-suppressive (NSP) fields at Avon and Minnipa yielded 89–244 pg of DNA g^−1^ soil of *R. solani* AG 8 (**Table 1**). These inoculum concentrations were considered within a high disease risk category based on southern Australian experience [65]. However, the analysis of root samples from 7 week old plants indicated significantly lower disease incidence in the SP field samples compared to those from the NSP fields, at both the sites (**Table 1**). *Rhizoctonia solani* AG8 is generally considered a seedling pathogen, hence root measurements were taken using seedlings 7 weeks after sowing in order to quantify differences between the two fields.

**Table 1:** Pathogen abundance and disease incidence.

Site	Field	*R. solani* AG 8 (pg DNA g^−1^ soil)	Root rating (0–5 scale)	% infected crowns
**Avon**	**Suppressive**	89±17 a	0.269±0.019 a	6.9±1.4 a
	**Non-supp**	244±25 b	3.181±0.132 c	59.6±4.0 b
**Minnipa**	**Suppressive**	107±32 a	0.444±0.036 a	8.4±1.4 a
	**Non-supp**	167±52 ab	2.181±0.148 b	59.0±4.3 b

Note: values within each column followed by the same letter are not statistically significant at P<0.05.

Amount of pathogen *R. solani* AG 8 inoculum in the surface soil at sowing and the level of disease incidence measured in 7 week old seedlings from suppressive and non-suppressive fields at Avon and Minnipa with standard errors.

**Figure 1:**
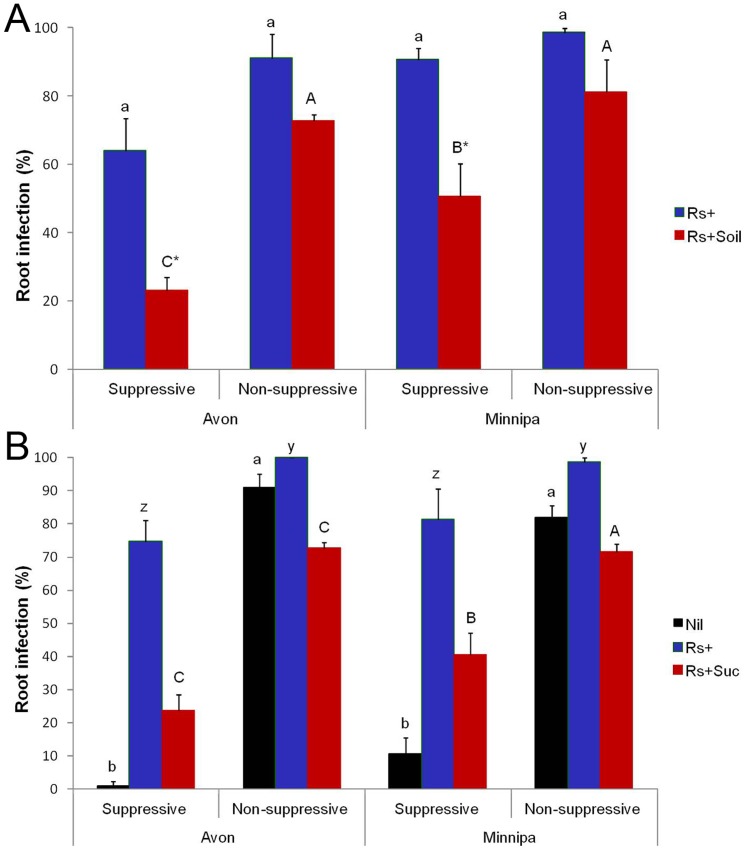
Rhizoctonia disease suppression in wheat seedlings. Results from a 28 day bioassay using soils collected from field sites at the start of the crop season in 2010. Soil transfer bioassay (A); sterile soil with *R. solani* AG 8 (Rs+, blue) inoculum and sterile soil + *R. solani* AG 8 (Rs+, red) inoculated with 10% of field soil. Bars of same color with different letters are significantly different at P<0.05 (ANOVA), * indicates significant reduction (P<0.008; ANOVA) in disease with field add soil addition. Disease suppression potential bioassay (B); field soil (Nil, black), field soil with *R. solani* AG 8 (Rs+, blue) and field soil +C added (Suc) + *R. solani* AG 8 (Rs+suc, red). Disease suppression potentials – 68.4 & 27.3 Avon soils; 50.1 & 27.6 Minnipa soils. Bars of similar color with different letters are significantly different at P<0.05 (ANOVA).

Results from the two growth chamber assays also clearly differentiated the level of disease suppression potential (DSP) in the soils from the two locations and indicated higher suppression potential in the soils from SP fields at both sites compared to NSP fields (**Figure 1A**). In the soil transfer assay, the addition of fresh soil from SP fields plus pathogen inoculum significantly (ANOVA, P<0.001) reduced disease incidence in sterile soil by 63.8% and 44.1% in the Avon and Minnipa soils, respectively, compared to addition of pathogen inoculum alone. In contrast, the addition of fresh soil from the NSP fields caused only a minor reduction in disease incidence in the sterile NSP soil (17.6–20.1%). In the DSP bioassay the addition of *R. solani* AG 8 inoculum increased disease levels considerably in all soils but the disease suppression potential was significantly (ANOVA, P<0.011) lower (mean = 27%) in the non-suppressive soil compared to suppressive soil (mean = 46%) (**Figure 1B**). A higher level of disease response to added inoculum coupled with a smaller reduction following C addition in NSP fields suggests a lower suppressive ability of these soils.

Compared to the suppressive soils, Avon non-suppressive soils showed higher levels of total fungal DNA both at sowing and at 7 weeks, but no difference in fungal DNA quantity was observed in the Minnipa soils (**Table S2**). A wider fungal to bacterial ratio was observed in the non-suppressive soil at sowing (62.8) compared to the suppressive soils (14.9). A reduction in the fungi:bacteria DNA ratio ‘in-crop’ was only seen in the Avon NSP soil but not in Minnipa soils or Avon SP soil. Although a measurable amount of *Trichoderma* spp. DNA was found in all soils (average = 56 pg±41 DNA/g soil) there was no trend based on soil type, suppression or sampling time. *Trichoderma* DNA levels were generally lower in the Minnipa soils compared to the Avon soils. PERMANOVA analysis of T-RFLP data (**Table 2**) showed significant effects at both sowing and in-crop for suppression that was supported by NMDS ordination (**Figure S2**).

**Table 2:** PERMANOVA analysis.

Dataset	Factor	Sowing		7 weeks	
		CV	P	CV	P
**t-RFLP**	**Site**	20.90	0.001	13.14	0.008
	**Supp**	16.20	0.002	10.70	0.033
	Site * Supp	21.50	0.003	18.50	0.006
**Whole Dataset**	**Site**	13.89	0.001	13.62	0.001
	**Supp**	12.63	0.001	7.36	0.001
	Site * Supp	13.93	0.001	13.56	0.002
**Resample Dataset**	**Site**	14.13	0.001	13.68	0.001
	**Supp**	12.90	0.001	7.61	0.004
	Site * Supp	14.23	0.001	13.93	0.001

Statistical comparisons of the t-RFLP, the 28S whole dataset, and the 28S re-sampled (4484 sequences per sample) datasets. CV = Component of variation.

### Fungal 28S LSU sequence processing and classification

Of 1,260,461 fungal 28S sequences, a total of 994,430 passed quality filtering (**Table S3**) that were then classified against the RDP fungal classifier reference database with a range between 4,484 and 44,431 reads per sample. Of these, 345,066 were read from the reverse (LR3) direction. Unless explicitly stated, all results are based on the re-sampled dataset at 4,484 reads per sample. Sequences classified as *Eukaryota incertae sedis* that encompassed non-fungal eukaryotes comprised 1.6±0.3% and 5.3±1.5% of reads in the Avon and Minnipa soils, respectively, and were removed prior to analyses. Rarefaction curves indicated near-coverage saturation in some samples, with no consistent differences in coverage among sampling locations (**Figure S3**).

**Figure 2:**
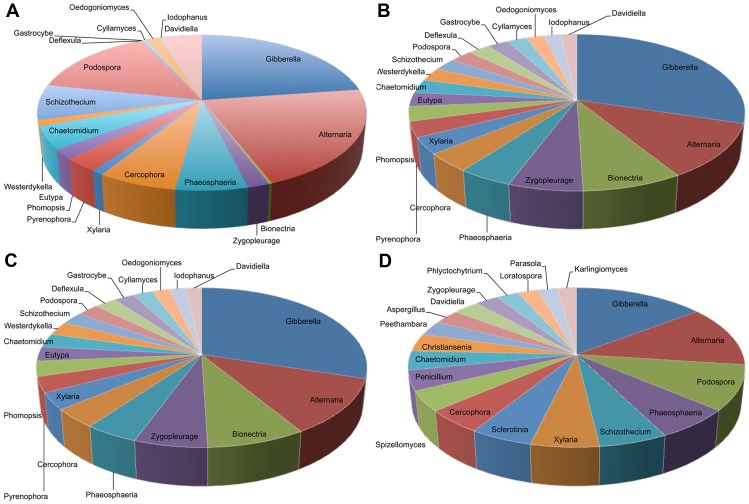
Composition of the 20 most abundant genera. Data from (A) suppressive soils, (B) non-suppressive soils, (C) Avon soil, and (D) Minnipa soil.

Best-match classification yielded a total of 917 unique genera that covered 54% of the RDP Fungal Classifier reference database. Ascomycota (78.1±9.0%), followed by Basidiomycota (9.5±4.7%), and Chytridiomycota (5.9±4.8%) dominated the fungal communities. Of the 37 fungal classes identified, the top 10 accounted for 95.5±2.8% of all sequences. Likewise, the top 10 of 104 orders contained 77.8±7.7% of all sequences, the top 20 of 298 families accounted for 75.7±6.9%, and the top 20 of 917 genera had 56.2±7.6% of all classified reads (**Figure 2**). Results and discussion of classification at 50% bootstrap confidence (**Text S2**) and differences between the whole dataset and the randomly resampled datasets (**Text S3**) are discussed in supplementary information.

### Suppressive versus Non-Suppressive Fungal Community Comparisons

**Figure 3:**
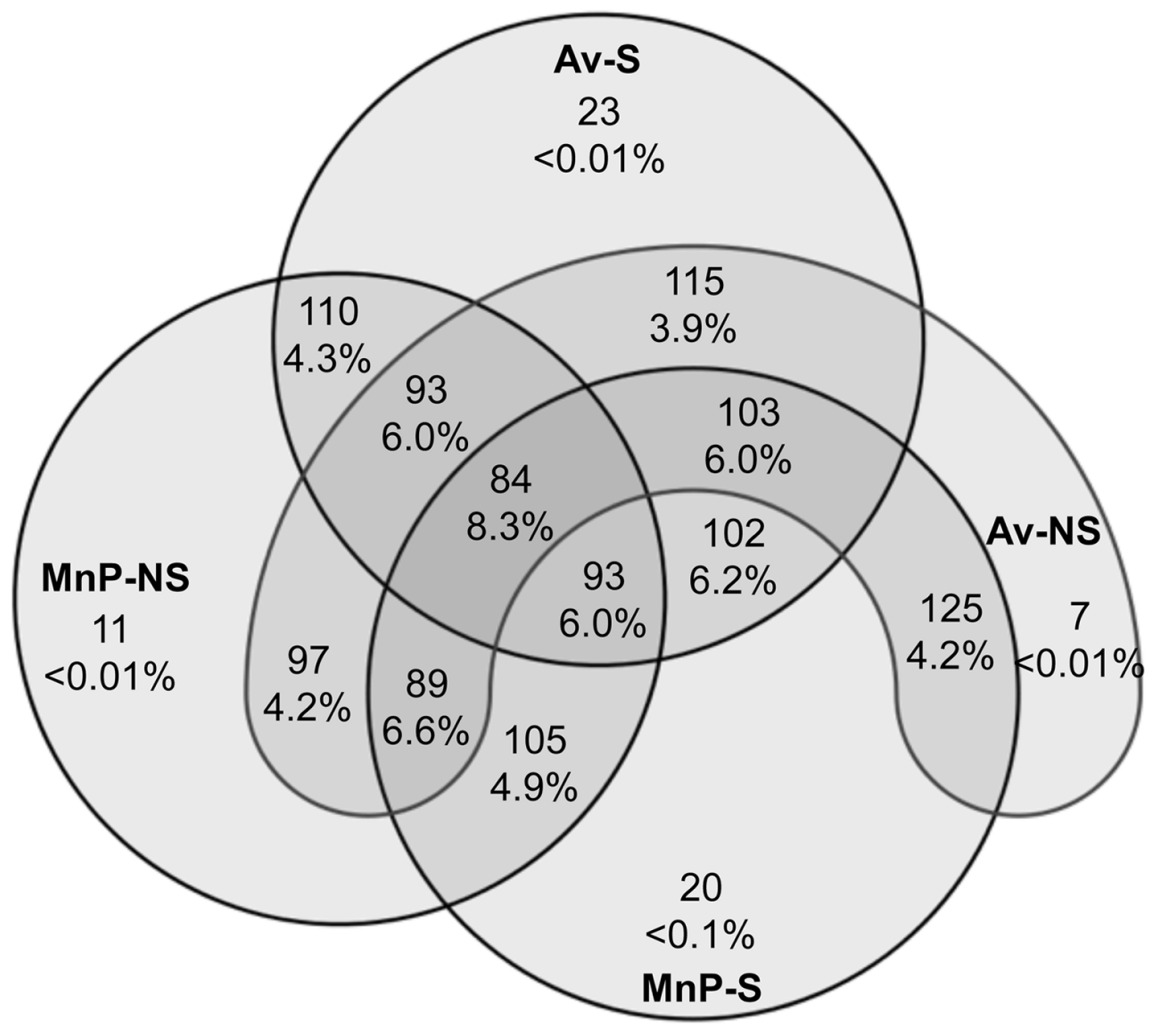
Venn diagram for genus-level OTUs. Number of shared and unique taxa at the genus level among four sites using the criterion that >60% of the replicates in a given sowing sample contain sequences belonging to that OTU. Percentages denote the proportion of shared reads over the total reads obtained and the sites are shown by their abbreviations. Overlapping areas indicate shared taxa among sites. Av-S (Avon suppression), Av-NS (Avon non-suppression), MnP-S (Minippa suppression), MnP-NS (Minippa non-suppression).

**Figure 4:**
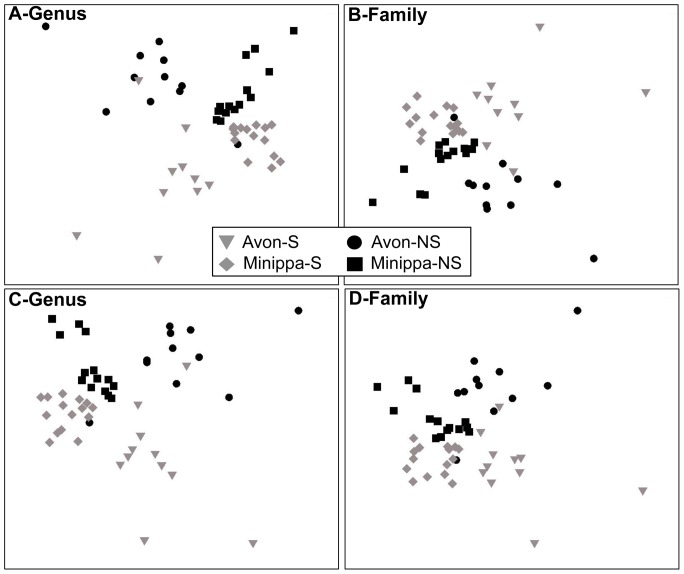
Non-metric dimensional scaling (NMDS) based ordinations for differences among sites and treatments. Analyses generated from Bray Curtis dissimilarity plus a dummy variable (+d) on Hellinger-transformed relative abundances for all-data at 0% bootstrap (closest match) at the genus (A) and family (B) levels and the re-sampled data at the genus (C) and family (D) levels. 2D stress values were 0.17 (A), 0.18 (B), 0.18 (C), and 0.19 (D).

**Figure 5:**
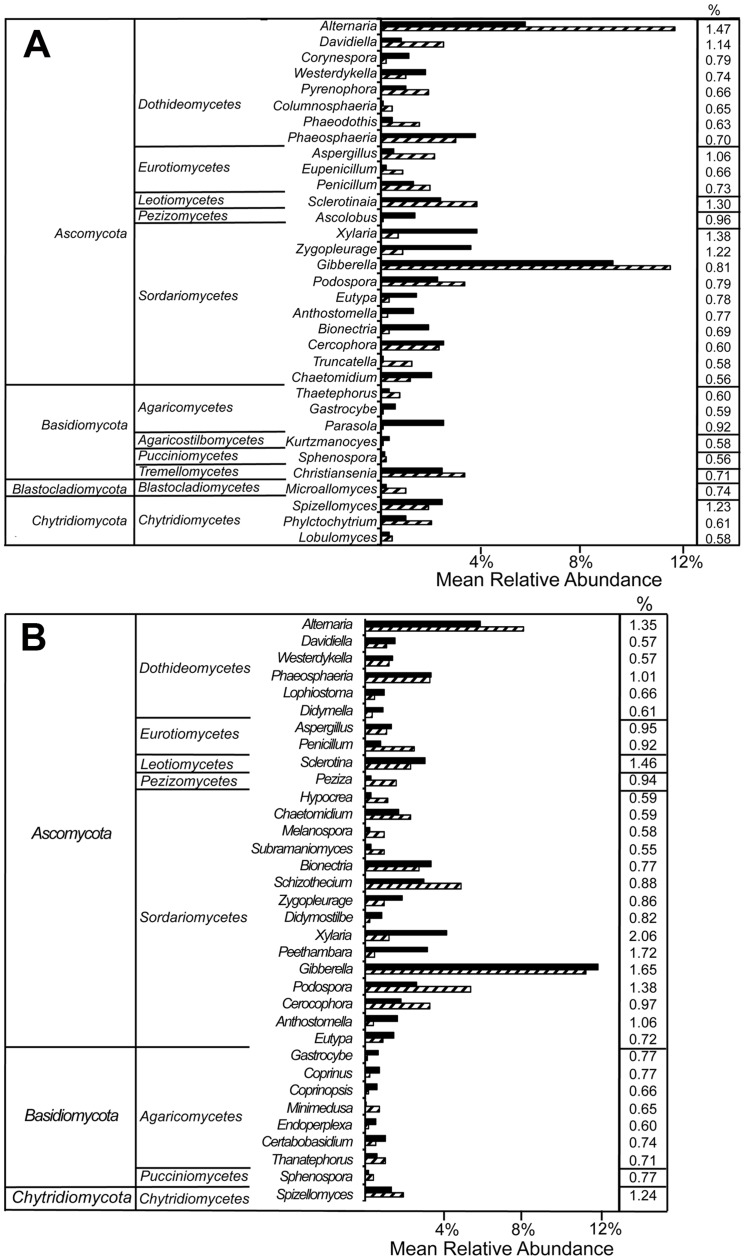
Similarity percentage analysis (SIMPER). Relative abundances (%) of OTUs at the genus level that contribute to the discrimination between fungal communities in suppressive and non-suppressive soils (solid bar = suppression, hatched bar = non-suppression) in the (A) sowing and (B) in-crop (7 week) samples. Numbers in the right column indicate percent contribution to discrimination by SIMPER analysis (sum = 26.8% (A) and 31.2% (B)). * indicate significant differences between suppressive and non-suppressive fields (t-test, p<0.05). Bold taxa indicate that they are shared between the sowing and in-crop samples.

Based on the criterion that >60% of the replicates per site must contain a certain genus-level OTU, we determined to what degree these OTUs were shared in the samples at sowing. A total of 84 OTUs, comprising 8.3% of all sequences, were shared among all sites (**Figure 3**). A very small proportion (<0.1%) of sequences were unique to any one location while suppressive and non-suppressive fields shared 102 and 97 genera, respectively. ANOSIM two-way crossed analysis showed significant differences in fungal community composition between both suppression status and soil type at the genus, family, and order units. Global R statistics decreased at higher taxonomic levels, suggesting a lower degree of separation between the different suppression groups (**Table 3**). PERMANOVA analysis indicated significant soil type effect for W (CV = 13.89, P = 0.001) and for RS (CV = 14.13, P = 0.001) (**Table 2**). Non-metric MDS ordination illustrated the dissimilarities in fungal communities at the best genus level matches among the four sites, with smaller differences at the family level (**Figure 4**). The average contribution of individual genera OTU to the overall Bray-Curtis distances (SIMPER analysis) showed that 33 genera were responsible for 26.8% and 31.2% of the discrimination between suppressive and non-suppressive soils at sowing and in-crop, respectively (**Figure 5**). No significant differences were identified in measures of Shannon diversity (H’) and Pielou’s evenness (*J’*) (**Table S2**).

**Table 3:** Soil type and suppression status statistical comparisons.

Group		Original dataset			Re-sampled dataset		
		Genus	Family	Order	Genus	Family	Order
**Soil Type**	**Global R**	0.542	0.511	0.294	0.558	0.497	0.288
	**p<**	0.01	0.01	0.01	0.01	0.01	0.01
**Suppression**	**Global R**	0.751	0.665	0.518	0.750	0.652	0.510
	**p<**	0.01	0.01	0.01	0.01	0.01	0.01

Significance of differences (main effects only) at different taxonomic levels using ANOSIM two-way crossed analysis for the original dataset and re-sampled dataset.

### Plant development

NMDS ordination showed a much stronger discrimination at sowing versus at 7 weeks (data not shown). This was also reflected in the PERMANOVA results, where the coefficient of variation decreased in the site, suppression, and site x suppression factors with time (**Table 2**). Only 28% of the top 33 genera significantly contributing to the suppressive community discrimination (based on SIMPER analysis at sowing and in-crop) were shared between the two time periods. The majority of those belonged to the class Sordariomycetes, where 56% of the genera were shared. In contrast, only one genus was shared among the 10 that comprised the Agaricomycetes between the sowing and in-crop periods. *Alternaria* significantly (t-test, p<0.01) decreased with time in the non-suppressive soils from 11.7% to 8.1% relative abundance, while *Schizothecium* increased (t-test, p<0.01) 3 to 7 fold to 3.0% and 4.8% of the community in the suppressive and non-suppressive soils, respectively. A large 8-fold decrease with time (t-test, P<0.01) was associated with *Christiansenia* in both soils. Both *Ascolobus* (t-test, P<0.01) and *Corynespora* (P<0.05) decreased from 1.3% to 0.5% and 1.1% to 0.3% relative abundance in the suppressive soils but significantly increased (t-test, P<0.05) three-fold in the non-suppressive soils. *Zygopleurage* decreased from 3.6% to 1.9% (t-test, P<0.05) only in the suppressive soil. Lastly, the *Hypocrea* increased with time (t-test, P<0.05) from 0.2% to 1.2% of the community in the non-suppressive soils with no increase in the suppressive sites.

## Discussion

### Suppressive versus non-suppressive fungal community discrimination

Significant fungal composition differences were revealed between suppressive and non-suppressive soils at each site as well as for both sites combined, which were also shown by the independent T-RFLP method. For example, according to the SIMPER results, 4.0% of the genera identified in this study accounted for 26.8% and 31.2% of the discrimination between suppressive and non-suppressive soils in the sowing and in-crop samples, respectively. A number of fungal genera with plant pathogen suppression potential were identified as well as fungi causing diseases of wheat, maize, and grapevines (**Table S4**). In this context we identify the average classification confidence (CC) and standard deviation for each genus discussed in order to provide insight into the dissimilarity between the query sequences and the reference database closest match. The inferred characteristics discussed are based on studied members of each genus, but there could be variation in phenotype within these genera that could affect suppressiveness or disease.

The genera most associated with the suppressive fields identified through the SIMPER analysis largely contained putative endophytes, saprophytes and fungi that may play a role in disease suppression (**Figure 5**). Differences were due mostly to changes in relative abundances, not presence/absence, as indicated by the similar number of shared and unique OTUs among treatments (**Figure 3**). In general, members of the Xylariaceae, Bionectriaceae and Hypocreaceae families with known antifungal capability are well represented among the dominant communities in the suppressive fields. For example, *Xylaria* (CC = 68±19%), a genus reported with primarily endophytic and some saprotrophic species [57], exhibited the largest gain (t-test, P<0.05) in abundance in the suppressive community of any genus. Endophytes such as these are known to stimulate plant growth, improve the ability of plants to withstand environmental stresses, and increase disease resistance [58]. Specifically, *Xylaria* has also been reported to exhibit antifungal activity against plant pathogenic fungi probably through secondary metabolites such as xylarinic acids [59]–[60]. The *Bionectria* (CC = 96±13%) that were also enriched, though not significantly, contains species that are saprotrophic, necrotrophic, or biotrophic. The genus also includes mycoparasites that are used as biocontrol agents of fungal plant pathogens [61]. Also higher (t-test, P<0.05) in suppressive soil were the *Eutypa* (CC = 77±20%), which include species responsible for grapevine dieback disease [62] and the *Anthostomella* (CC = 50±14%), which are primarily saprotrophic [63]. Some other fungi that trended towards higher abundance in suppressive soils with reported antifungal activity include *Chaetomium*, *Corynascus* and *Microdiplodia* spp. [64]–[66].

In contrast to the suppressive fields, the majority of putative fungal pathogens had higher abundances in the non-suppressive fields. *Gibberella* (CC = 90±14), whose anamorph is *Fusarium* sp., the cause of crown rot of wheat, a common disease in Australian wheat fields was found in higher abundance, though not significantly, in the non-suppressive fields. This genus may also include non-pathogenic isolates of *Fusarium oxysporum*, identified as a biocontrol agent [67]–[68]. *Alternaria* (CC = 93±15%), the causative endophytic fungus for a leaf blight and black point of wheat, which has also been routinely isolated as an endophyte of leaves from maize and wheat [69] was associated with non-suppressive (P<0.05) soils. It is the most abundant endophyte in wheat cultivars [70] and in maize [71]. Likewise, *Podospora* (CC = 58±16%) and *Penicillium* (CC = 95±13%) were associated (P<0.05) with the non-suppressive soil, in contradiction to another study involving pea crop soil in Denmark where they were associated with healthy soils [35]. *Aspergillus* sp. (CC = 62±15%), and *Penicillium* sp. are endophytes isolated from wheat leaves and roots [70], [72] and dominate in the non-suppressive soils. *Thanatephorus* (CC = 97±10%), the telomorph of the genus *Rhizoctonia*, which are saprotrophic and disease-causing endophytes, were found in slightly higher numbers in non-suppressive soils. Two other genera that contain wheat pathogens, *Pyrenophora* (cause of tan spot) and *Phaeosphaeria* (cause of glume blotch), did not significantly change between fields.

### Plant development

In addition to the pathogenic fungi, the rhizosphere environment would be dominated by fungi capable of responding rapidly to carbon substrates from rhizodeposition (r-selection) whereas the fungal community in the bulk soil depends on a diverse array of carbon substrates. Therefore, the rhizosphere fungal community composition likely differs from the bulk soil [73]–[74]. Fungal communities at sowing represent the bulk soil while the 7-week (in-crop) communities also reflect the rhizosphere-induced changes [26], as seen from the change in fungal community structure shown in Figure 5. The build-up of *R. solani* AG 8 inoculum in wheat and other cereals during this early plant growth period was previously observed [75] and we also observed an increase in *R. solani* in both the suppressive and non-suppressive soils, although the increase was greater in diseased fields. *R. solani* AG 8 is an effective saprophyte hence may grow in the rhizosphere in SP fields. Despite changes in the fungal composition from sowing to 7 weeks, fungal genera with potential to contribute to antibiosis, mycoparasitism and mycorrhization remained the dominant members in the SP communities, suggesting the importance of a fungal contribution to the stability of the disease suppression capacity. Plants can exploit their interaction with rhizosphere bacterial and fungal communities for protection against infection, plant growth promotion and can also benefit from an induced resistance [26], [30], [76]. The importance of the variable response by the putative plant pathogens from sowing to 7 weeks requires further investigation.

### Effect of soil characteristics on the fungal community

Variation in the soil physico-chemical properties and the spatial and temporal distribution of plant residues can have a significant impact on the diversity and biomass of fungal communities. Soil type is similar at both our field sites, but there was some difference in the soil texture (% clay) especially between the SP and NSP fields. Although there was significant variation in the fungal diversity based on soil type (PERMANOVA analysis), the effect was smaller than that associated with suppression capacity. Crop and soil management (tillage) practices, which influence the quantity and quality of organic carbon sources, could also influence populations and biomass of specific fungi and thus modify diversity over time [77]–[78]. Land use and soil pH have been shown to influence the diversity of some fungal groups [79] but the effect of pH on fungi was weaker than that on bacterial diversity [80]. In one study Hypocreales made up to 5% of fungi in soils with high pH (8.0) while they were absent in soils with pH below 5.5 [80]. Members of the Hypocreales (e.g. *Hypocrea, Bionectrecia* and *Peethambara*) were also part of the dominant group of fungi in the alkaline pH soils at both sites we studied. Organic C and total N levels were consistently higher in the soils from SP fields at both sites mainly from the long-term (>10 y) adoption of stubble retention and reduced till cropping practices compared to <5 y for these practices in the NSP fields. Above and below ground plant residues from the annual crops are the only source of C inputs (<1 tonne C per annum) hence the stocks of decomposable particulate organic matter are lower (<30% of total C) than those generally found in tundra grassland and forest soils [81]–[82]. Overall, the observation of soil type based differences in fungal community composition illustrates the need for the assessment of multiple soil types and locations in order to decipher the signature(s) of a disease suppressive microbial community(s).

### Diversity profiling of suppressive and non-suppressive soils

Direct classification of ∼1 million filtered sequences yielded 917 genera. After re-sampling and normalizing for read abundance, our genus-level richness was equivalent to or higher than other studies that used 28S pyrosequencing with clustering [83]–[84] or even with high throughput ITS sequencing [35], [73], [77], [85], [86] which resolves at species level. While such direct comparisons are difficult due to sample size effects, clustering cut-offs and relating 28S to ITS data, as a group they do indicate that diversity in these alkaline, calcareous, low organic matter soils is higher than might be expected. This may be partially attributed to the use of a 125 g soil sample for DNA extraction, since, the larger than typical (<1 g) sample size reduces small-scale spatial heterogeneity thereby decreasing replicate variability while encompassing a diverse array of microhabitats. Ophel-Keller [41] had previously noted the need for large soil samples to accurately determine changes in soilborne pathogenic fungi and relate them to disease risk categories in the field. Conversely, the high diversity may also be attributed to the soil physico-chemical properties, quality and quantity of organic plant residues and environmental factors (e.g. low rainfall) that may favor soil fungi with their ability to access large volumes of soil through hyphal networks.

The dominance of Ascomycota in these cropping soils where organic matter is concentrated primarily at the surface is similar to that found in tundra soils [87] whereas Basidiomycota was the most abundant phylum in forest soils [77], [88]. Rarefaction curves were nearly saturated in some samples, indicating that approximately 20,000 sequences was sufficient to cover the majority of genus-level diversity with diminishing returns thereafter. This saturation is highly ecosystem and depth-dependent, with near-saturation occurring at less than 4,000 sequences in permafrost soils at depth (40–50 cm) [49].

### Disease suppression

The level of impact of soil-borne disease incidence on plant growth is influenced by the amount of plant pathogen present and its interactions with the plant, soil and environmental factors. A main criterion defining biological disease suppression is the ability of soil biotic communities to suppress the disease expression in the host plants even in the presence of adequate virulent inoculum and a susceptible host. *R. solani* AG 8 levels measured at sowing indicate the presence of sufficient inoculum to cause significant disease [89]. Differences in pathogen inoculum levels between suppressive and non-suppressive soils were small, indicating similar pathogen pressure. Field disease assessments showing distinct differences in the disease levels in the SP and NSP fields validate the suitability of these field soils to investigate disease suppressive communities. These field results are supported by the differences in the DS potential of suppressive and non-suppressive soils. Grain yields of crops were higher (avg. >30%) in the suppressive fields compared to non-suppressive fields confirming better plant performance partly due to the lack of disease in the SP fields.

The biological disease suppression observed at these field sites is effective against multiple soil-borne pathogens (16) and under multi-crop rotations. Hence we hypothesize that both the general and specific disease suppression may be acting simultaneously. Continued supply of crop residue carbon stimulates general microbial activity including fungal populations that can compete with *R. solani* for resources [18], [22], [90] and also may increase the build-up of antibiotic producing/antagonistic microflora including fungi [19]. TRF profiling and sequence data both show differentiation of fungal communities of SP and NSP soils from both field sites but the combined lack of consistent trends in the total fungal biomass (measured as DNA concentration) suggests that the change in community composition may play an important role in the observed suppression. Since a number of pathogenic fungi can become active saprophytes depending upon resource availability and host presence, total fungal biomass may not truly reflect the net ecological role of entire soil fungal community. The significant difference (PERMANOVA) in the fungal community structure in the soils from 7 weeks, representing the root microbiome suggests a relationship between the disease incidence (root health) and associated fungal community. While we found that the fungal component of soil communities was different in suppressive versus their paired non-suppressive soil, bacteria and their interactions with fungi and plants could also be important to biological disease suppression [13], [30], [91], and is the subject of continuing work for these soils.

## Conclusions

In a comparative analysis of soils with high and low suppressive potential, from the wheat-cropping region in South Australia, we measured the fungal community composition using targeted pyrosequencing of the 28S LSU gene. Overall, the study clearly demonstrates the need to include soil fungal communities, along with bacterial based investigations, in order to obtain a full understanding of disease suppressive community. The observation of significant fungal community composition differences between SP and NSP soils prior to sowing suggest varying pathogen-fungal community interactions in the suppressive and non-suppressive soils, whereas the differences observed at 7 weeks demonstrate a significant relationship between the fungal community structure and root health. The dominance of members of the order Hypocreales with known antifungal capability in the suppressive soils highlights the importance of pathogenic fungi - general fungal community interactions in the disease suppression.

The taxonomically based assessment of fungal community structure should also be strengthened with functional characterization at species level resolution, in order to decipher the mechanisms of disease suppression. In this context, the ITS region would be preferred and thus, the development of a robust and taxonomically concise ITS-based fungal classifier is needed. Lastly, findings from this type of sequencing based study could be used to direct future isolation and manipulation studies both to understand the mechanisms of disease suppression and to develop disease control options.

## Supporting Information

Figure S1 **Examples of field and root conditions from non-suppressive fields.** (A) Avon non-suppressive field at 16 weeks post-sowing, (B) roots from 2 week-old samples from the Avon non-suppressive field, (C) roots from the Avon suppressive field at 2 weeks.
(TIF)

Figure S2 **Non-metric dimensional scaling (NMDS) of ITS rRNA-T-RFs.** Ordination based on Bray Curtis similarity plus a dummy variable (+d) with square root transformation of ITS rRNA-T-RFs from (A) sowing and (B) in crop (7 wk) sampling. 2D stress 0.14 (A) and 0.17 (B).
(TIF)

Figure S3 **Rarefaction curves for genus-level bins.**
(TIF)

Figure S4 **Relationship between the proportions of unclassified reads at each taxonomic level and suppression status.** Data shows averages and standard errors.
(TIF)

Figure S5 **Classification confidence (bootstrap) for all genera within classes.** Data based on classes that contain >0.5% read abundance from RDP Fungal Classifier. The cumulative percent of total sequences is denoted by the graph on the right.
(TIF)

Figure S6 **Non-metric dimensional scaling (NMDS) with all unclassified removed and only unclassified reads.** Ordination based on Bray Curtis similarity plus a dummy variable (+d) with Hellinger-transformed relative abundances for all-data at 50% bootstrap at the genus level with all unclassified removed (A) and with only the unclassified reads (B). 2D stress values were 0.17 (A), 0.21(B). NS =  non-suppressive soil, S =  suppressive soil.
(TIF)

Figure S7 **Correlation between original and re-sampled dataset.** Relative genera OTU abundance between the original dataset and the re-sampled dataset.
(TIF)

Table S1 **Soil characteristics of the Avon and Minippa sites.** Standard errors shown. Supp = suppressive and Non-supp = non suppressive, CEC = cation exchange capacity.
(DOCX)

Table S2 **Summary of results from 28S sequencing and T-RFLP profiling data.** Standard deviations are shown adjacent to means and Fisher’s least significant difference test results are presented in the bottom row.
(TIF)

Table S3 **Summary of sequence processing results using the Ribosomal Database Project (RDP) pyrosequencing pipeline.** Sequencing read information with the number of raw pyrosequencing reads, processed sequences and average processed sequence length. Filters include: Primer trimmer allowing 0 mismatches, N count filter, length >400 bp filter and exponential quality filter (Q>20). Site abbreviations: Av – Avon, Min-Minippa, S-suppression, NS-non-suppression, Sow-sowing, IC-in-crop samples at 7 wks post sowing.
(DOCX)

Table S4 **Primary habitat, function and disease suppression related property for dominant fungal genera**.
(TIF)

Text S1 **Fungal 28S amplification and sequence processing.**
(DOCX)

Text S2 **Unclassified fungal diversity.**
(DOCX)

Text S3 **Comparisons of resampled to whole 28S sequence dataset.**
(DOCX)
