# Supplementary figures and images for: Fungal Community Structure in Disease Suppressive Soils Assessed by 28S LSU Gene Sequencing
Source: PLoS One. 2014 Apr 3;9(4):e93893. doi: 10.1371/journal.pone.0093893 (PMC3974846; doi:10.1371/journal.pone.0093893)

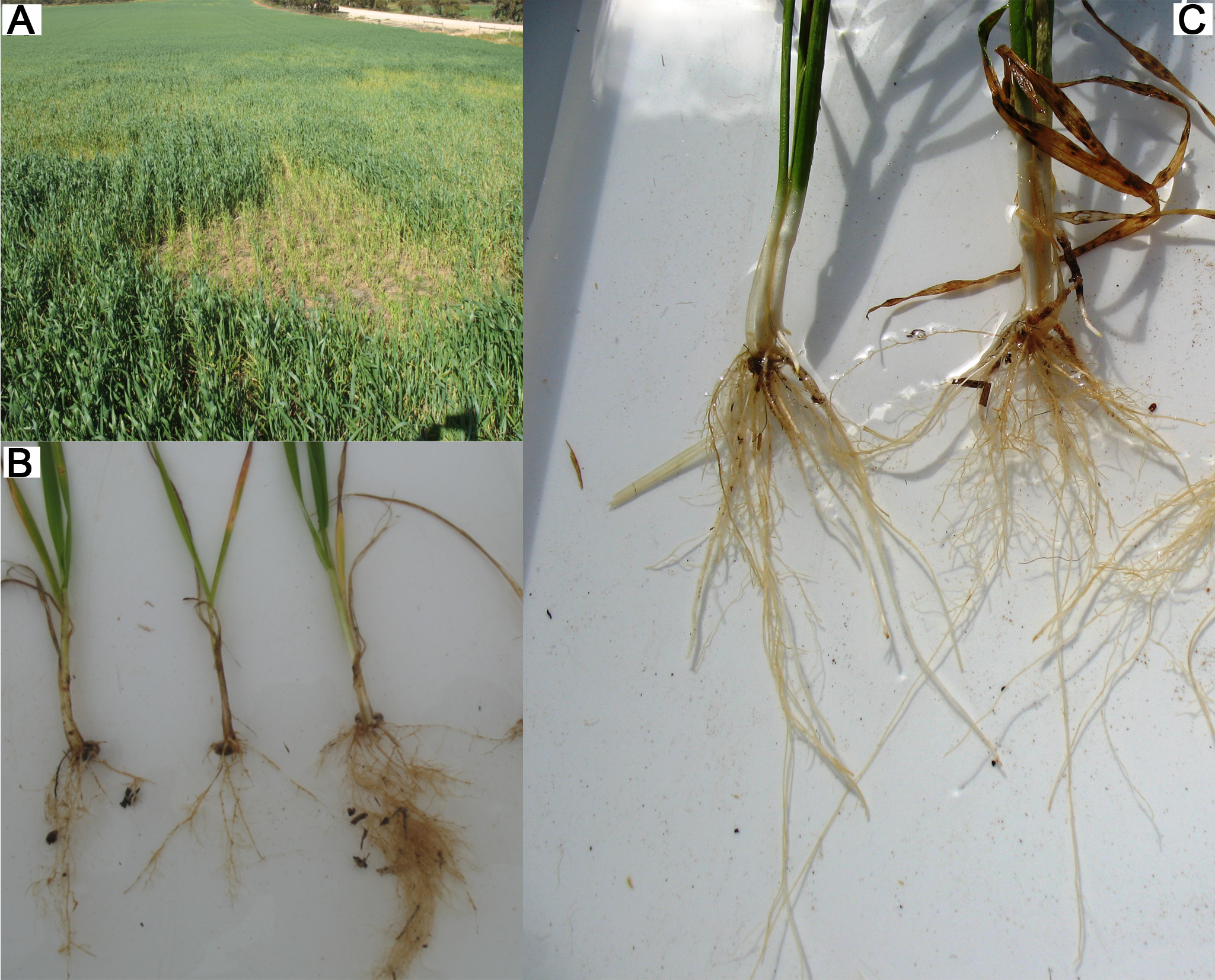

Supplement: Figure S1 — Examples of field and root conditions from non-suppressive fields. (A) Avon non-suppressive field at 16 weeks post-sowing, (B) roots from 2 week-old samples from the Avon non-suppressive field, (C) roots from the Avon suppressive field at 2 weeks. (TIF) [file pone.0093893.s001.tif]

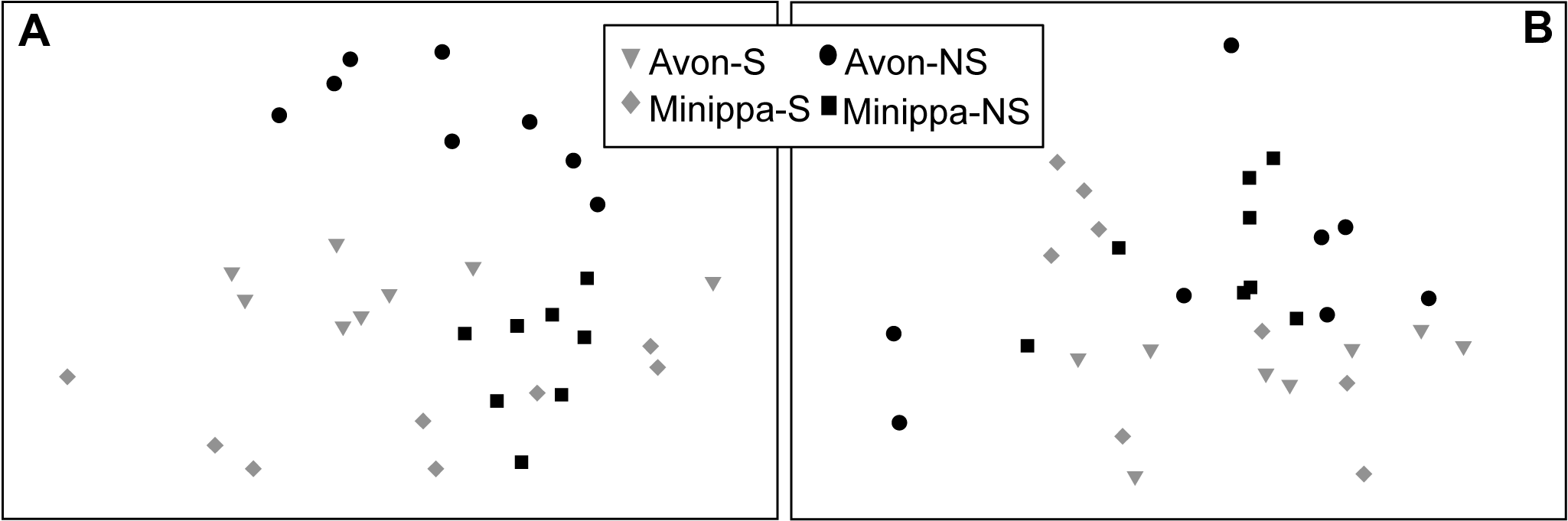

Supplement: Figure S2 — Non-metric dimensional scaling (NMDS) of ITS rRNA-T-RFs. Ordination based on Bray Curtis similarity plus a dummy variable (+d) with square root transformation of ITS rRNA-T-RFs from (A) sowing and (B) in crop (7 wk) sampling. 2D stress 0.14 (A) and 0.17 (B). (TIF) [file pone.0093893.s002.tif]

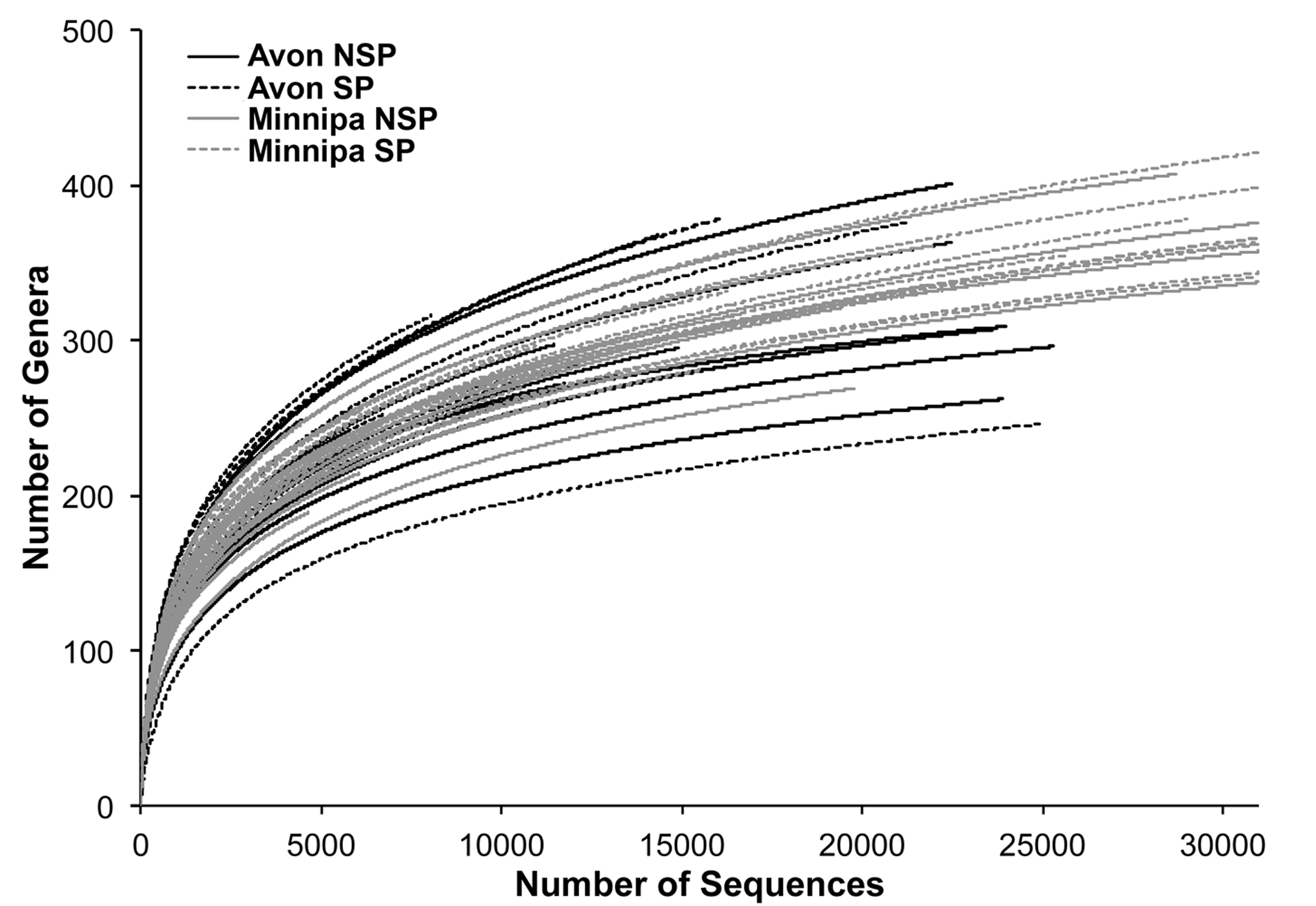

Supplement: Figure S3 — Rarefaction curves for genus-level bins. (TIF) [file pone.0093893.s003.tif]

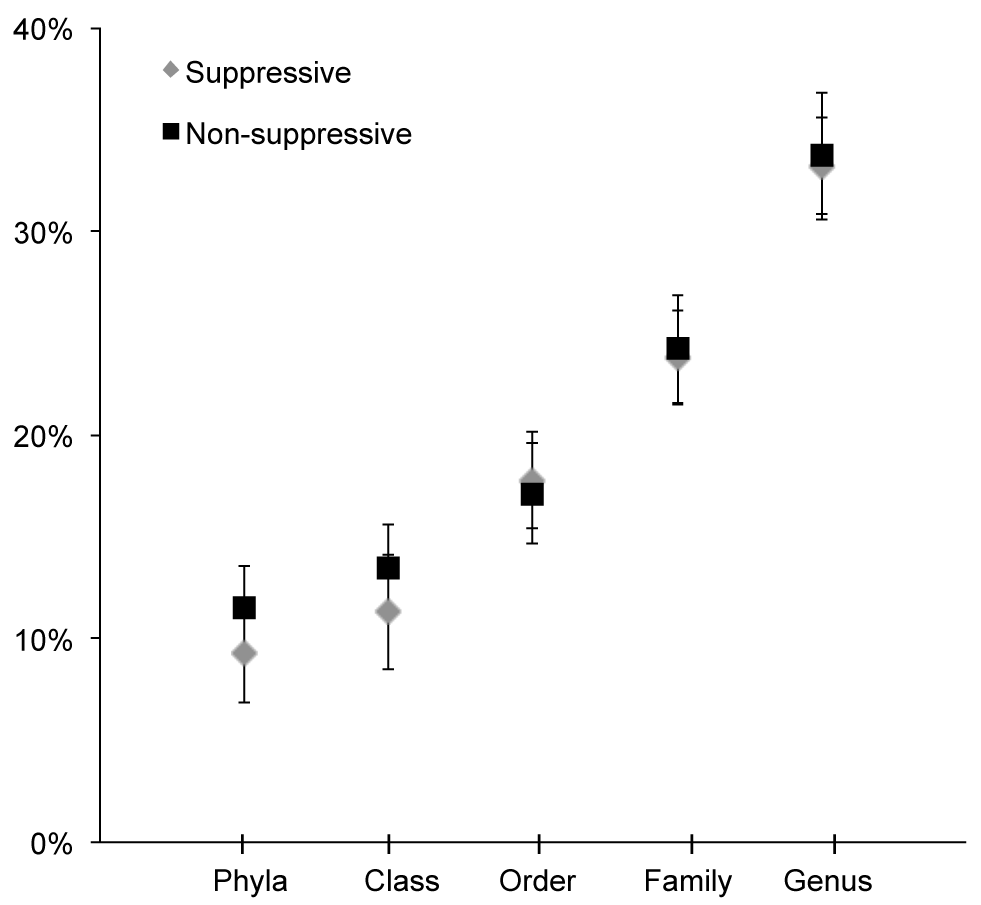

Supplement: Figure S4 — Relationship between the proportions of unclassified reads at each taxonomic level and suppression status. Data shows averages and standard errors. (TIF) [file pone.0093893.s004.tif]

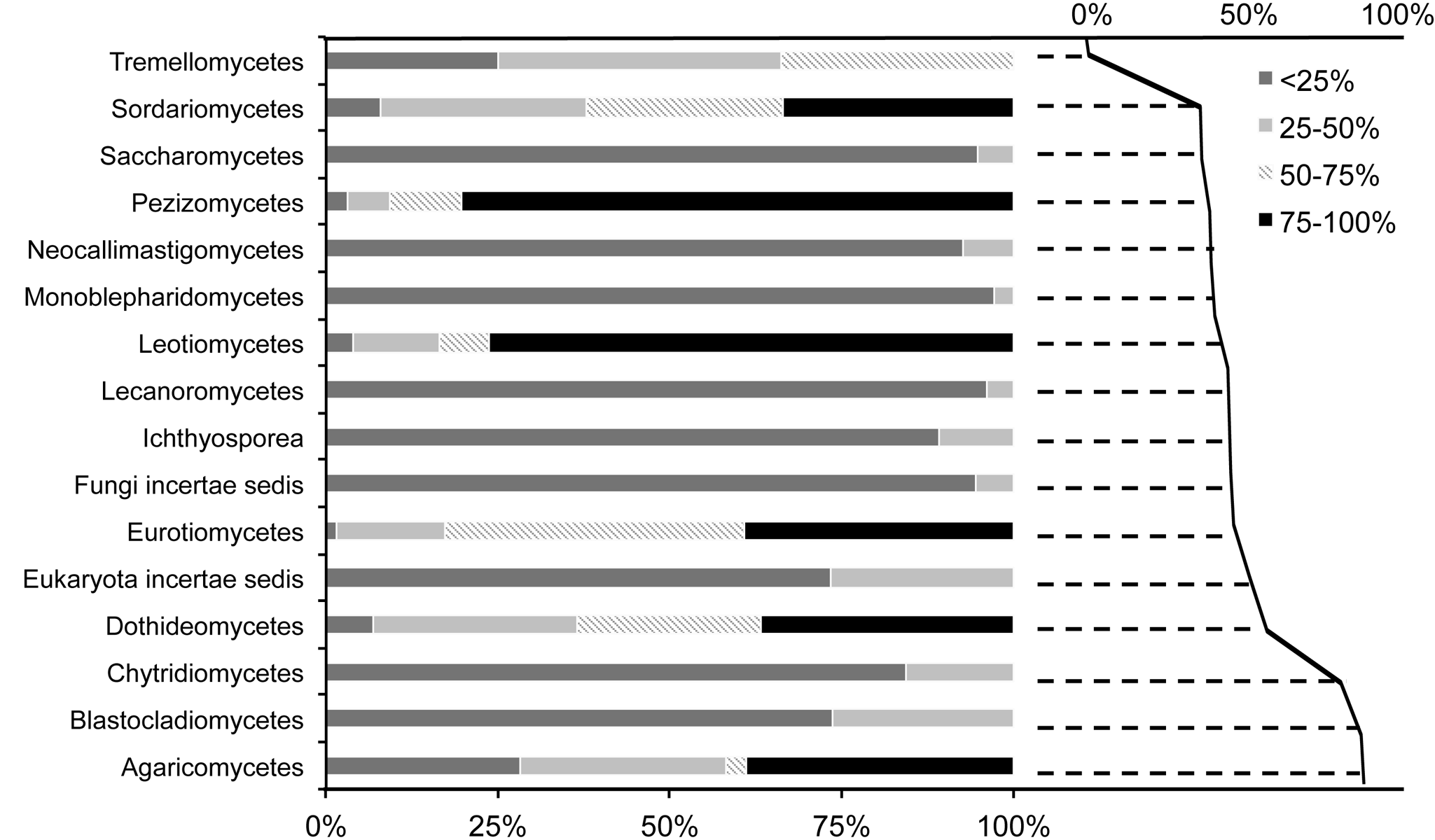

Supplement: Figure S5 — Classification confidence (bootstrap) for all genera within classes. Data based on classes that contain >0.5% read abundance from RDP Fungal Classifier. The cumulative percent of total sequences is denoted by the graph on the right. (TIF) [file pone.0093893.s005.tif]

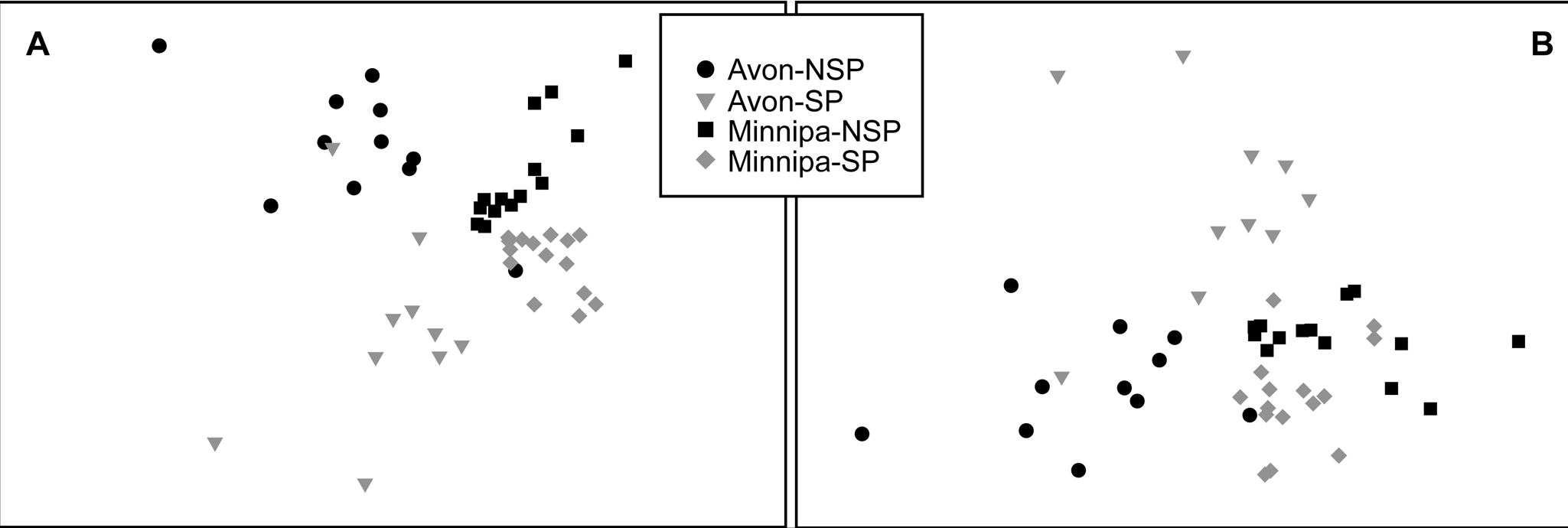

Supplement: Figure S6 — Non-metric dimensional scaling (NMDS) with all unclassified removed and only unclassified reads. Ordination based on Bray Curtis similarity plus a dummy variable (+d) with Hellinger-transformed relative abundances for all-data at 50% bootstrap at the genus level with all unclassified removed (A) and with only the unclassified reads (B). 2D stress values were 0.17 (A), 0.21(B). NS = non-suppressive soil, S = suppressive soil. (TIF) [file pone.0093893.s006.tif]

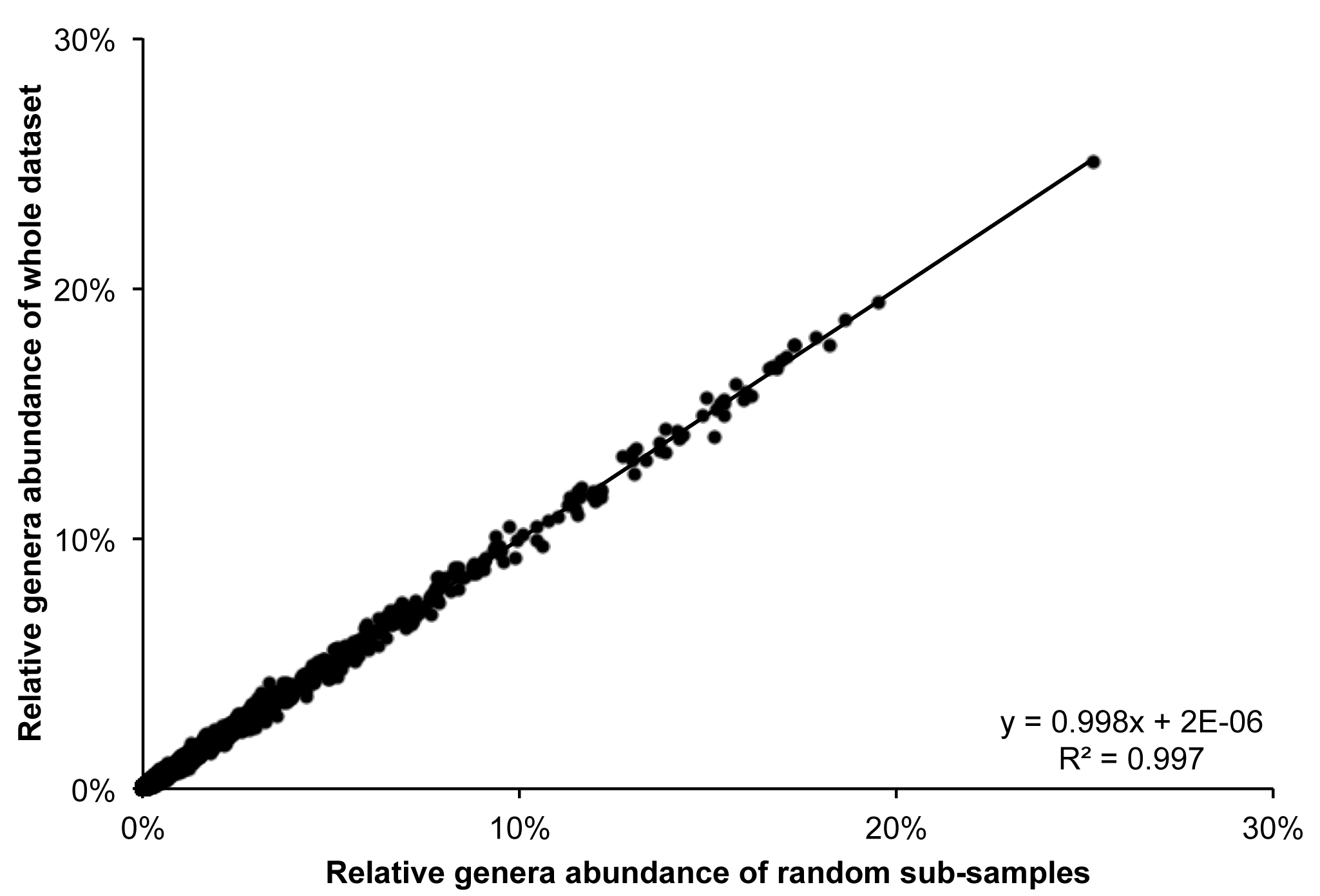

Supplement: Figure S7 — Correlation between original and re-sampled dataset. Relative genera OTU abundance between the original dataset and the re-sampled dataset. (TIF) [file pone.0093893.s007.tif]

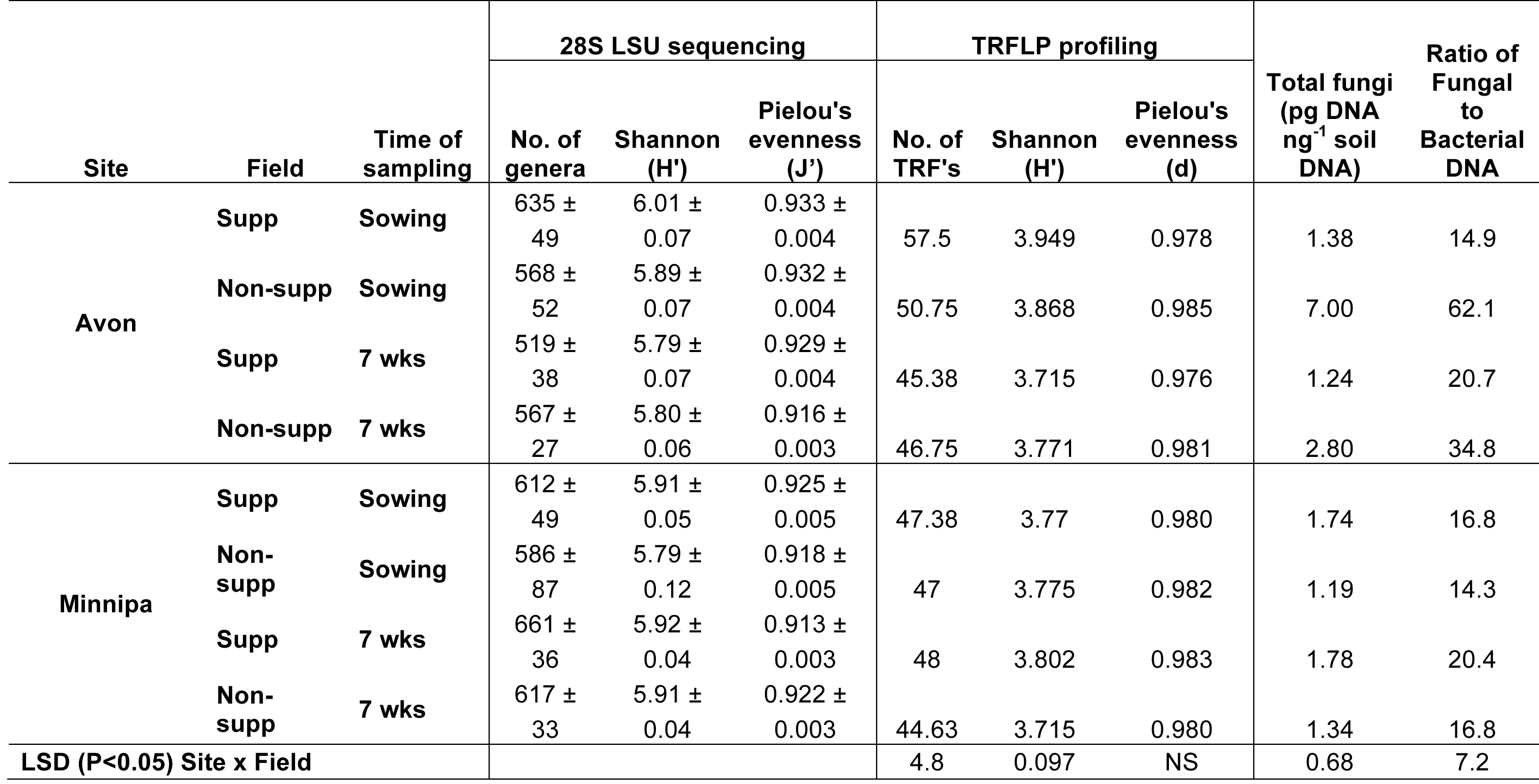

Supplement: Table S2 — Summary of results from 28S sequencing and T-RFLP profiling data. Standard deviations are shown adjacent to means and Fisher’s least significant difference test results are presented in the bottom row. (TIF) [file pone.0093893.s009.tif]

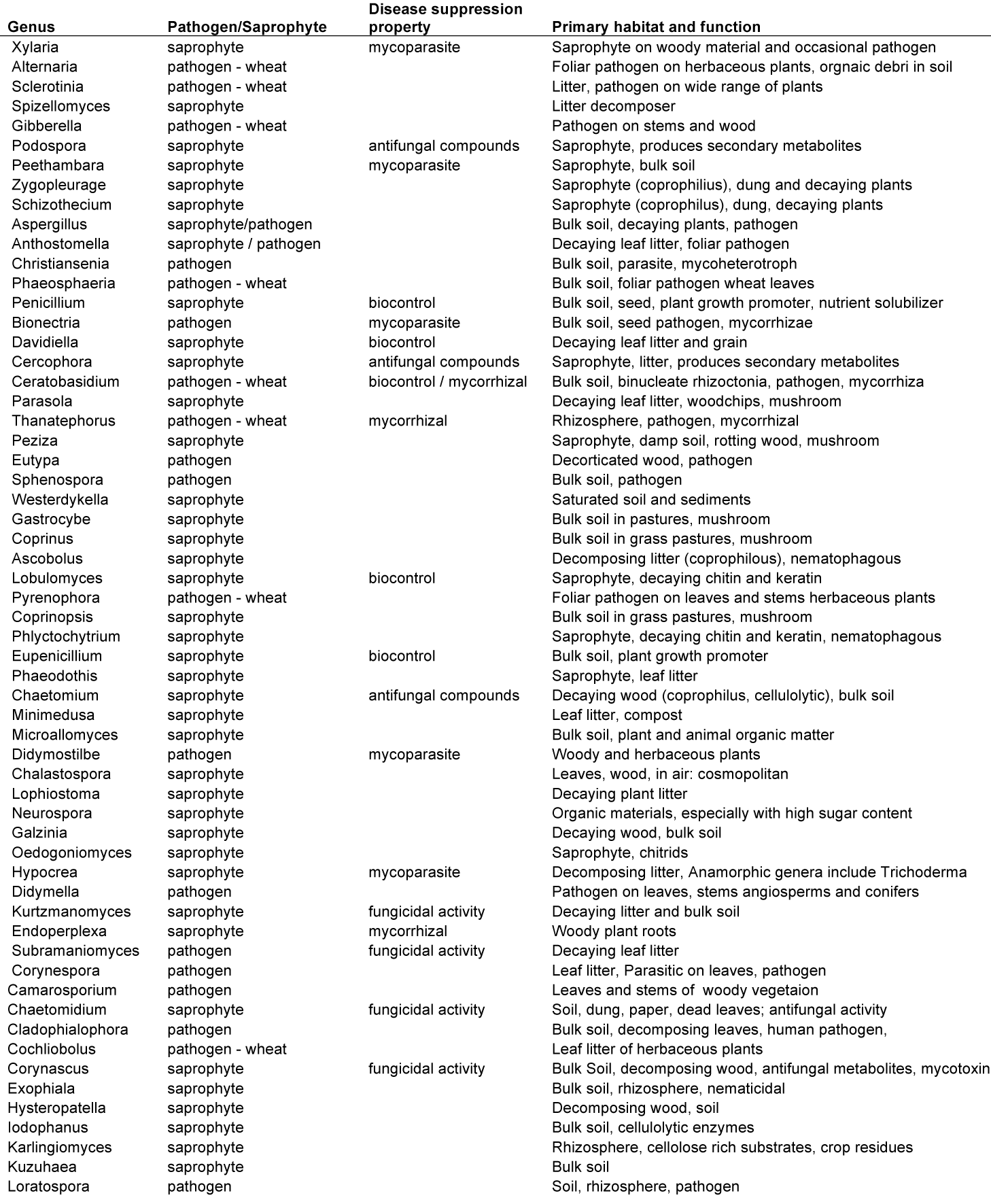

Supplement: Table S4 — Primary habitat, function and disease suppression related property for dominant fungal genera. (TIF) [file pone.0093893.s011.tif]
