# Supplementary material for: Fungal Community Structure in Disease Suppressive Soils Assessed by 28S LSU Gene Sequencing
Source: PLoS One. 2014 Apr 3;9(4):e93893. doi: 10.1371/journal.pone.0093893 (PMC3974846; doi:10.1371/journal.pone.0093893)
Supplement: Table S1 — Soil characteristics of the Avon and Minippa sites. Standard errors shown. Supp = suppressive and Non-supp = non suppressive, CEC = cation exchange capacity. (DOCX) [file pone.0093893.s008.docx]

| **Site** | **Field** | **Organic C** | **Total N** | **pH** | **CEC** | **Clay** | **Silt** | **Sand** | **C:N ratio** |
| --- | --- | --- | --- | --- | --- | --- | --- | --- | --- |
|  |  | % | |  | meq/100g | % | | |  |
| **Avon** | **Supp** | 1.60±0.09 | 0.15±0.02 | 8.3±0.27 | 19.8±1.0 | 17.2±0.3 | 31.8±5.9 | 51.1±5.7 | 10.7 |
|  | **Non-supp** | 0.79±0.12 | 0.07±0.01 | 8.1±0.08 | 3.65±0.5 | 10.9±0.9 | 21.8±2.8 | 67.3±2.3 | 11.8 |
| **Minnipa** | **Supp** | 1.15±0.11 | 0.12±0.03 | 8.4±0.14 | 14.0±1.3 | 17.1±3.2 | 21.8±2.1 | 61.2±2.9 | 9.6 |
|  | **Non-supp** | 1.04±0.11 | 0.09±0.02 | 8.3±0.17 | 20.9±1.3 | 12.5±1.8 | 14.3±0.9 | 56.4±2.0 | 11.6 |
