# Supplementary material for: Fungal Community Structure in Disease Suppressive Soils Assessed by 28S LSU Gene Sequencing
Source: PLoS One. 2014 Apr 3;9(4):e93893. doi: 10.1371/journal.pone.0093893 (PMC3974846; doi:10.1371/journal.pone.0093893)
Supplement: Table S3 — Summary of sequence processing results using the Ribosomal Database Project (RDP) pyrosequencing pipeline. Sequencing read information with the number of raw pyrosequencing reads, processed sequences and average processed sequence length. Filters include: Primer trimmer allowing 0 mismatches, N count filter, length >400 bp filter and exponential quality filter (Q>20). Site abbreviations: Av – Avon, Min-Minippa, S-suppression, NS-non-suppression, Sow-sowing, IC-in-crop samples at 7 wks post sowing. (DOCX) [file pone.0093893.s010.docx]

**Table S3. Summary of sequence processing results using the Ribosomal Database Project (RDP) pyrosequencing pipeline.** The RDP pipeline initial process parameters were as follows: Forward primer max edit distance=2, maximum number of N’s=0, minimum sequence length=400, minimum read Q (exponential quality) score=20.

| **Sample** | **Site** | **Raw Seqs** | **Filtered Seqs** | **Seq Length** | **Primer Trimmer** | **N count >0** | **>400 Length Filter** | **Exp Quality Filter** |
| --- | --- | --- | --- | --- | --- | --- | --- | --- |
| **1** | **Av-S-Sow** | 2228 | 1699 | 506 | 2 | 132 | 318 | 77 |
| **2** | **Av-S-Sow** | 9619 | 8044 | 489 | 19 | 377 | 740 | 439 |
| **3** | **Av-S-Sow** | 3796 | 2975 | 496 | 4 | 126 | 617 | 74 |
| **4** | **Av-S-Sow** | 19222 | 16039 | 497 | 40 | 770 | 1596 | 777 |
| **5** | **Av-S-Sow** | 22879 | 14402 | 504 | 20 | 1268 | 4988 | 2201 |
| **6** | **Av-S-Sow** | 8156 | 6740 | 512 | 9 | 306 | 908 | 193 |
| **7** | **Av-S-Sow** | 24604 | 21243 | 497 | 18 | 1392 | 689 | 1262 |
| **8** | **Av-S-Sow** | 4288 | 3250 | 507 | 8 | 201 | 706 | 123 |
| **9** | **Av-NS-Sow** | 4238 | 3356 | 495 | 0 | 143 | 628 | 111 |
| **10** | **Av-NS-Sow** | 2892 | 2302 | 515 | 9 | 143 | 396 | 42 |
| **11** | **Av-NS-Sow** | 2320 | 1687 | 502 | 3 | 122 | 424 | 84 |
| **12** | **Av-NS-Sow** | 5528 | 4484 | 491 | 14 | 251 | 533 | 246 |
| **13** | **Av-NS-Sow** | 14787 | 11451 | 504 | 11 | 806 | 2020 | 499 |
| **14** | **Av-NS-Sow** | 18303 | 14909 | 509 | 13 | 703 | 2273 | 405 |
| **15** | **Av-NS-Sow** | 15063 | 11753 | 507 | 26 | 735 | 2108 | 441 |
| **16** | **Av-NS-Sow** | 31183 | 22546 | 502 | 42 | 2129 | 5177 | 1289 |
| **17** | **Min-S-Sow** | 31443 | 25734 | 526 | 36 | 1247 | 3560 | 866 |
| **18** | **Min-S-Sow** | 34589 | 28726 | 517 | 43 | 1215 | 3689 | 916 |
| **19** | **Min-S-Sow** | 21437 | 16258 | 509 | 32 | 1203 | 3032 | 912 |
| **20** | **Min-S-Sow** | 13502 | 10949 | 520 | 22 | 461 | 1657 | 413 |
| **21** | **Min-S-Sow** | 9136 | 6740 | 509 | 14 | 535 | 1516 | 331 |
| **22** | **Min-S-Sow** | 7710 | 6148 | 518 | 5 | 329 | 1088 | 140 |
| **23** | **Min-S-Sow** | 45784 | 37350 | 498 | 36 | 1756 | 4521 | 2121 |
| **24** | **Min-S-Sow** | 4224 | 2976 | 507 | 6 | 257 | 797 | 188 |
| **25** | **Min-NS-Sow** | 8078 | 6044 | 505 | 18 | 402 | 1203 | 411 |
| **26** | **Min-NS-Sow** | 23996 | 19814 | 497 | 36 | 1033 | 2017 | 1096 |
| **27** | **Min-NS-Sow** | 6091 | 4910 | 496 | 7 | 203 | 878 | 93 |
| **28** | **Min-NS-Sow** | 2501 | 1877 | 509 | 3 | 135 | 419 | 67 |
| **29** | **Min-NS-Sow** | 2800 | 2213 | 517 | 4 | 132 | 391 | 60 |
| **30** | **Min-NS-Sow** | 1689 | 1111 | 506 | 2 | 107 | 506 | 63 |
| **31** | **Min-NS-Sow** | 58851 | 44431 | 508 | 82 | 3511 | 8203 | 2624 |
| **32** | **Min-NS-Sow** | 51977 | 37569 | 503 | 47 | 3698 | 8508 | 2155 |
| **33** | **Av-S-IC** | 31390 | 24843 | 503 | 39 | 1124 | 4551 | 733 |
| **34** | **Av-S-IC** | 12711 | 9335 | 508 | 10 | 589 | 2410 | 367 |
| **35** | **Av-S-IC** | 13972 | 11448 | 513 | 10 | 566 | 1667 | 281 |
| **36** | **Av-S-IC** | 7152 | 5784 | 517 | 10 | 238 | 958 | 162 |
| **37** | **Av-S-IC** | 16623 | 12139 | 506 | 23 | 967 | 2658 | 836 |
| **38** | **Av-S-IC** | 1649 | 1288 | 519 | 4 | 77 | 247 | 33 |
| **39** | **Av-S-IC** | 562 | 380 | 499 | 0 | 18 | 155 | 9 |
| **40** | **Av-S-IC** | 159 | 111 | 511 | 0 | 6 | 40 | 2 |
| **41** | **Av-NS-IC** | 632 | 470 | 510 | 2 | 41 | 107 | 12 |
| **42** | **Av-NS-IC** | 660 | 492 | 512 | 0 | 28 | 123 | 17 |
| **43** | **Av-NS-IC** | 29800 | 22476 | 506 | 37 | 1805 | 4147 | 1335 |
| **44** | **Av-NS-IC** | 16225 | 13186 | 513 | 29 | 630 | 1948 | 432 |
| **45** | **Av-NS-IC** | 29231 | 23603 | 497 | 44 | 1215 | 3763 | 606 |
| **46** | **Av-NS-IC** | 29916 | 24035 | 516 | 34 | 1332 | 3981 | 534 |
| **47** | **Av-NS-IC** | 29816 | 23907 | 515 | 40 | 1434 | 3632 | 803 |
| **48** | **Av-NS-IC** | 30479 | 25331 | 516 | 54 | 1119 | 3282 | 693 |
| **49** | **Min-S-IC** | 22262 | 15555 | 502 | 26 | 1550 | 4151 | 980 |
| **50** | **Min-S-IC** | 13104 | 10637 | 510 | 17 | 501 | 1682 | 267 |
| **51** | **Min-S-IC** | 31276 | 24937 | 498 | 32 | 1222 | 4424 | 661 |
| **52** | **Min-S-IC** | 49466 | 40291 | 519 | 39 | 2099 | 6146 | 891 |
| **53** | **Min-S-IC** | 49969 | 41095 | 517 | 22 | 2162 | 5618 | 1072 |
| **54** | **Min-S-IC** | 48788 | 40772 | 518 | 52 | 1924 | 4937 | 1103 |
| **55** | **Min-S-IC** | 51530 | 39640 | 514 | 76 | 2577 | 7560 | 1677 |
| **56** | **Min-S-IC** | 35448 | 29043 | 514 | 40 | 1405 | 4120 | 840 |
| **57** | **Min-NS-IC** | 40467 | 32141 | 498 | 46 | 1633 | 5743 | 904 |
| **58** | **Min-NS-IC** | 24282 | 19446 | 517 | 26 | 1130 | 3214 | 466 |
| **59** | **Min-NS-IC** | 13868 | 11250 | 514 | 9 | 667 | 1655 | 287 |
| **60** | **Min-NS-IC** | 8341 | 6713 | 515 | 13 | 368 | 1071 | 176 |
| **61** | **Min-NS-IC** | 46771 | 35565 | 509 | 50 | 2769 | 6972 | 1415 |
| **62** | **Min-NS-IC** | 44415 | 36306 | 512 | 43 | 1754 | 5277 | 1035 |
| **63** | **Min-NS-IC** | 30481 | 21993 | 502 | 38 | 2047 | 5207 | 1196 |
| **64** | **Min-NS-IC** | 20740 | 16675 | 515 | 25 | 836 | 2861 | 343 |

Sequencing read information with the number of raw pyrosequencing reads, processed sequences and average processed sequence length. Filters include: Primer trimmer allowing 0 mismatches, N count filter, length >400 bp filter and exponential quality filter (Q>20). Site abbreviations: Av – Avon, Min-Minippa, S-suppression, NS-non-suppression, Sow-sowing, IC-in-crop samples at 7 wks post sowing.
