# Supplementary material for: Fungal Community Structure in Disease Suppressive Soils Assessed by 28S LSU Gene Sequencing
Source: PLoS One. 2014 Apr 3;9(4):e93893. doi: 10.1371/journal.pone.0093893 (PMC3974846; doi:10.1371/journal.pone.0093893)
Supplement: Text S1 — Fungal 28S amplification and sequence processing. (DOCX) [file pone.0093893.s012.docx]

**Fungal 28S amplification and sequence processing**

Quadruplicate 20 μl PCR reactions were performed as follows: 4 μl Promega GoTaq buffer, 0.5 μl GoTaq DNA polymerase, 1.5 μl Roche 25 mM MgCl_2_, 1 μl Invitrogen 10 mM dNTP mix, 1 μl of each primer (10 pmol μl^-1^), 0.2 μl New England BioLabs 10 mg ml^-1^ BSA, 1 μl 10 ng μl^-1^ template, and 9.8 μl H_2_O. Cycling conditions were 94°C for 3 min followed by 30 cycles of 94°C for 1 min, 51°C for 40 s, 72°C for 1 min, followed by an extension at 72°C for 10 min. Replicates were pooled and gel purified using the Qiagen Gel Purification Kit following band excision and purified using the Qiagen PCR purification kit. Amplicons were sequenced following adapter ligation by Utah State University CIB Genomics Core Lab using Lib-L kits and processed using the shotgun protocol.

Raw sequences were quality processed using the RDP pyrosequencing pipeline initial process tool. Parameters were as follows: Forward primer max edit distance=2, number of N’s=0, minimum sequence length=400, minimum read Q (exponential quality) score=20. Samples were sorted according to their tag identifier sequence.
