# Supplementary material for: Fungal Community Structure in Disease Suppressive Soils Assessed by 28S LSU Gene Sequencing
Source: PLoS One. 2014 Apr 3;9(4):e93893. doi: 10.1371/journal.pone.0093893 (PMC3974846; doi:10.1371/journal.pone.0093893)
Supplement: Text S2 — Unclassified fungal diversity. (DOCX) [file pone.0093893.s013.docx]

**Unclassified fungal diversity**

The composition of the unclassified fungal lineages was investigated by implementing a 50% bootstrap classification confidence (CC) threshold using the RDP Fungal Classifier (rdp.cme.msu.edu/classifier). Classifications were binned as “unclassified” until a confidence of at least 50% was reached at a given higher taxonomic level. These defined “unclassified” bins occur at various taxonomic ranks; an unclassified sequence at the genus level may remain classified at coarser taxonomic levels. The RDP fungal database (release 1) currently contains a total of 8,506 sequences: 2,395 Ascomycota, 6024 Basidiomycota, 51 Chytridiomycota, 16 Glomeromycota, 4 *Eukaryota incertae sedis*, and 5 Fungi incertae sedis. Therefore we may expect that a greater number of unclassified reads would be binned to under-represented taxa. However, there was no correlation between database taxon coverage and the proportion of unclassified reads. The proportion of unclassified reads decreased from 33.7±7.4% of all genera to 10.5±5.7% of all phyla (**Figure S4**). The range of confidences was also not evenly distributed among genera. The classes with the lowest classification confidence of their member genera were the *Lecanoromycetes*, *Icthyosporea*, *Fungi incertae sedis*, *Eukaryota incertae sedis, Chytridiomycetes*, *Saccharomycetes,* and *Blastocladiomycetes* (**Figure S5).** The *Chytridiomycetes* exhibited the highest gross number of sequences with classification confidence percentages less than 50%. With an average sequence length of 506 bp, the average confidence to the genus level from the LR3 (66±32%) and the LR0R (64±30%) primers were similar. There was no correlation (r=-0.016) between read length and classification confidence.

In order to test the influence of unclassified bins on community data, we replicated analyses using the 50% bootstrap confidence genus-level dataset where all unclassified reads were removed and a dataset where only the unclassified reads were used. NMDS ordination shows that the removal of unclassified reads had some influence on the relationships between treatments and sites (**Figure S6**). Basing the ordination only on unclassified reads still preserved much of the NMDS structure, although the Minnipa suppression and non-suppression samples were less distinguishable (**Figure S6**). Significant site*treatment differences were found when only the unclassified sequences were used (PERMANOVA, F=6.34, P=0.001) and when the unclassified reads were removed (PERMANOVA, F=7.39, P=0.001).

The lack of correlation of confidence levels with RDP fungal database coverage shows that even the more “well-characterized” fungal lineages remain in need of further study. This was evident in many of our high-abundance classes such as the Dothideomycetes, Eurotiomycetes, Sordariomycetes and Agaricomycetes. Among the most unclassified phyla were members of the mostly aquatic Chytridiomycota, represented by 50 sequences that comprised 32 genera in the RDP database. The differences in the percent relative abundances of the unclassified bins at all taxonomic levels indicates the presence of a currently unknown but significant and dynamic proportion of the community that may contribute to suppression potential or compositional differences due to soil type and environment. The composition of the SIMPER dataset showed little change at 50% bootstrap, except for the inclusion of unclassified bins of the same classified genera in the list.

To date, the composition of the unclassified fungi has only been addressed at the coarser resolutions of the phylum and domain taxonomic levels by using BLAST-based methods [1]. Another study found that 71.5% of ITS sequences lacked an explicit taxonomic annotation with the use of an unfiltered reference dataset that dropped to 11% by forcing assignment to a classified nearest neighbor [2]. Another study showed that 68% of fungal OTUs could not be assigned to the order level [3]. In our study, the fact that only 34% of sequences could not be classified to the genus level is likely due to our samples originating in better studied agricultural soils as other less studied locations have shown to range up to 96% unclassified genera using identical methods [4].

**Supplementary References**

1. Lentendu G, Zinger L, Manel S, Coissac E, Choler P, Geremia RA, Melodelima C (2011) Assessment of soil fungal diversity in different alpine tundra habitats by means of pyrosequencing. Fungal Divers 49: 113–123.

2. Buée M, Reich M, Murat C, Morin E, Nilsson RH, Uroz S, Martin F (2009) 454 pyrosequencing analyses of forest soils reveals unexpectedly high fungal diversity. New Phytol 184: 449–456.

3. Artz RRE, Anderson IC, Chapman SJ, Hagn A, Schloter M, Potts JM, Campbell CD (2007) Changes in fungal community composition in response to vegetational succession during the natural regeneration of cutover peatlands. Microb Ecol 54:508-522.

4. Penton CR, St. Louis D, Cole JR, Luo Y, Wu L, Schuur EAG, Zhou J, Tiedje JM (2013) Fungal diversity in permafrost and tallgrass prairie soils under experimental warming conditions. Appl Environ Microb 79: 7063-7072.
