# Supplementary material for: Fungal Community Structure in Disease Suppressive Soils Assessed by 28S LSU Gene Sequencing
Source: PLoS One. 2014 Apr 3;9(4):e93893. doi: 10.1371/journal.pone.0093893 (PMC3974846; doi:10.1371/journal.pone.0093893)
Supplement: Text S3 — Comparisons of resampled to whole 28S sequence dataset. (DOCX) [file pone.0093893.s014.docx]

**Sequence resampling effects**

Random re-sampling to counter the variability in the number of sequences obtained per sample is being utilized more frequently in downstream analysis of large DNA datasets. Assumptions that re-sampled data accurately reflects the whole dataset are usually made without documented comparisons. Here we demonstrated that the relative abundances from both the whole and res-sampled dataset are highly correlated (R^2^=0.997; **Figure S7**) and the conclusions drawn from either sample set are statistically identical (**Table 2**) (**Table 3**). The relationship between NMDS sample ordination in both datasets was highly similar, despite some samples having >50,000 reads, compared to a re-sampled size of 4484 (data not shown). The choice of resemblance matrix and raw abundance transformation used for downstream analysis may influence the robustness of this relationship by changing the importance of rare OTUs. Here the use of a Bray-Curtis matrix with a dummy variable (+1) followed by Hellinger transformation reduces the influence of rare OTUs. The removal of all singletons resulted in nearly identical PERMANOVA and ANOSIM results for site, suppression and site*suppression interactions (data not shown).
